# Supplementary material for: Five days of heat acclimation improves cardiovascular and thermoregulatory responses without altering renal stress biomarkers in endurance athletes
Source: PLoS One. 2026 Aug 11;21(8):e0345346. doi: 10.1371/journal.pone.0345346 (PMC13460581; doi:10.1371/journal.pone.0345346)
Supplement: Supplementary file 1 [file pone.0345346.s002.docx]

Reviewer #1: Snape et al. investigate the effects of a 5-day heat acclimation (HA) protocol on cardiovascular and thermoregulatory responses, alongside renal stress biomarkers, in endurance-trained individuals. The study appears to be methodologically sound overall and addresses a relevant applied physiology question. However, I have several concerns regarding the framing of the hypotheses and the interpretation of the renal outcomes. In particular, the rationale underpinning the renal hypotheses is not fully convincing, and the null findings are, at times, overstated. The comments below are intended to help improve the clarity, balance, and physiological grounding of the manuscript.

Minor Comments

Introduction

“Improved plasma volume and sympathetic modulation following HA may preserve renal blood flow…”
Please provide a citation to support this statement. It is not clear that HA would preferentially preserve renal blood flow during exercise heat stress. If anything, the combination of heat stress and exercise is typically associated with renal vasoconstriction and reduced renal perfusion. The physiological basis for this statement requires further justification.

We thank the reviewer for this important observation. As they correctly point out, during exercise heat stress, renal blood flow is reduced by vasoconstriction associated with renal sympathetic nerve activation and vasopressin release (Chapman et al., 2021). We have reframed this statement to focus more directly on reduced risk of tubular injury and AKI risk, associated with lower core body temperature and better fluid regulation, linked to our collection of plasma NGAL and copeptin.

“These cardiovascular adaptations could offset the renal hypoperfusion…”
At present, the Introduction does not convincingly support the idea that HA could meaningfully offset reductions in renal blood flow during exercise in the heat. This point would benefit from either stronger supporting evidence or more cautious phrasing.

As stated above, we have rowed back on the argument of reduced renal blood flow, due to insufficient evidence to support this mechanism. Instead we have pivoted to reduced risk of tubular injury associated with hypovolemia and lower core body temperature.

“there influence…”
Typographical error: should read “their influence”.

We thank the reviewers for this observation and have corrected the text to state ‘their’.

“the kidneys exhibited an increased sensitivity to hyperthermia…”
This statement is too broad. Please clarify what is meant by “increased sensitivity” and specify the physiological evidence supporting this claim.

We once again thank the reviewers for this comment. This refers to the strong relationship between changes in serum creatinine and core temperature (hyperthermia) and not plasma NGAL (marker of renal ischaemia) in the study bv Connor et al., 2025. We have updated the paragraph to reflect the increased sensitivity is linked to ‘hyperthermia’ and not renal ischaemia.

The hypothesis that HA would attenuate renal tubular injury biomarkers appears insufficiently justified. You cite Connor et al., who used a longer HA protocol and reported no effect on NGAL, yet still hypothesise an attenuation with a shorter intervention. Please provide a clearer mechanistic or empirical rationale for this expectation.

We thank the reviewers for this important comment and agree further clarification is warranted. We have cited that the limitations in using fixed intensity protocols, such as those by Connor et al., 2025, evidenced by no change in plasma volume. This key adaptation may have reduced the level of hyperthermia and renal ischemia to the kidney (lower plasma NGAL). Although our study was shorter in length, it included a controlled hyperthermia protocol which have shown to be time efficient and effective strategies for lower thermal strain and inducing plasma volume expansion. Our study also included a broader panel of biomarkers, providing a more integrated view of systemic and renal responses to heat stress.

Methods

Heat stress test (HST):
You refer to gastrointestinal temperature (Tgi) but later mention rectal temperature (Tre). Please clarify which measure was used and ensure consistency throughout.

We have updated the text to report Tgi (gastrointestinal temperature), we thank the reviewer for noticing this error.

Blood sampling timing:
Please provide more detail on the timing of blood sampling during the HSTs, particularly the “post” sample. Was this obtained immediately post-exercise or after a delay? This is important for interpretation of biomarker responses.

We have corrected this text to state ‘immediately’ post exercise.

TEMP intervention description:
The TEMP condition is described as being performed at a “matched workload,” but it is unclear what this corresponds to (2.5 W/kg?). Given that the HOT condition is prescribed relative to core temperature, additional clarification is needed to allow comparison between conditions.

We thank the reviewer for this observation. The workload was matched at 2.5 W/kg for both groups, with this exercise intensity sufficient to elicit a core body temperature of 38.5 oC within 30-45 minutes, as suggested by Gibson et al., (2015). Further analysis showed no differences in heart rate and power output between groups over the 5-days suggesting matched cardiovascular strain and relative external workload. We have updated the text to specify the matched workload of 2.5 w.kg but not around HR and PO to save on word count, but will include if reviewer feels necessary.

Discussion

“reductions in neurohumoral markers… may have helped keep blood vessels in the kidneys more open…”
This statement would benefit from supporting evidence or a clearer mechanistic explanation. At present, the link between changes in these markers and renal vascular conductance during exercise heat stress is not well established.

We have provided further mechanistic explanation; although there is a lack of scientific evidence we have tried to summarise insights from the review by Chapman et al., 2021 on kidney pathophysiology to heat stress. These are framed as suggestions, with further research warranted. We are happy to retract this statement if the reviewer feels necessary.

Heat training and renal stress (Rønnestad et al., 2021)
This section appears somewhat inconsistent with the earlier hypothesis that HA may attenuate renal stress. If HA is proposed to be protective, the suggestion that repeated heat exposure could increase renal risk requires clearer reconciliation.

We have removed this sentence – we appreciate it is confusing in the context of the study hypothesis.

“no change observed… may reflect an underpowered design”
This statement is not fully convincing in its current form. Given that renal biomarkers represent a key novel aspect of the study, it would have been appropriate to base sample size calculations on expected changes in these variables. As prior literature (including studies cited by the authors) has reported similar null findings, this possibility should have been considered more explicitly in the study design and discussed more transparently here.

We thank the reviewer for this comment and agree that sample size considerations specific to renal biomarkers warrant explicit discussion. The study was powered a priori based on expected changes in core body temperature following 5 days of heat acclimation, which represented the most robust and well-characterised outcome available at the time of study design. Although renal responses were a central focus of the investigation, there were limited prior data quantifying the magnitude and variability of acute changes in blood biomarkers such as NGAL, KIM-1, copeptin, and normetanephrine in this experimental context, which precluded a reliable a priori power calculation for these variables. However, the absence of observable changes is consistent with several prior investigations; likely reflecting a limited physiological perturbation rather than a type II error.

Conclusion

“…longer HA protocols may be required… which could confer greater protection against renal injury…”
Based on the current data, this statement appears somewhat speculative. While it is reasonable to suggest that longer protocols may induce a more complete heat-adapted phenotype, the implication that this would translate to renal protection is not clearly supported by the findings presented. Consider tempering this statement or more clearly framing it as a hypothesis for future work.

We have reframed this to extended protocols (>10 days) to align with literature that has utilised protocols over 14 from occupational settings that also suggests the integration of hydration/cooling protocols (Schlader et al., 2019).

Figures

Consider including the outcomes of the two-way ANOVAs directly on the figures (e.g., main effects and interactions). This would improve readability and reduce the need to cross-reference figure captions.

The authors acknowledge this as an excellent idea – however, the software that we have used to produce the figures prohibits adding this to the figure. Nevertheless all interactions and main effects are highlighted in the figure title, and there was an absence of any significant interactions to report.

Reviewer #2: This manuscript investigates whether five days of short-term isothermic heat acclimation influences renal stress biomarkers and neuroendocrine responses during exercise-heat stress in endurance-trained athletes. The topic is timely and relevant given the increasing concern regarding exercise-associated kidney stress during training and competition in hot environments. The inclusion of renal tubular biomarkers (NGAL and KIM-1), alongside copeptin and normetanephrine, is a notable strength that increases the study's novelty. The study design is generally appropriate, and the authors successfully demonstrated classical heat acclimation adaptations, including reduced resting heart rate, plasma volume expansion, and increased sweat loss. Several important methodological and interpretative concerns should be addressed before the manuscript can be considered for publication.

Major Comments
1. Statistical power for biomarker outcomes
The sample size calculation appears to have been based primarily on thermoregulatory variables rather than renal biomarkers. Given the substantial inter-individual variability in responses to NGAL, KIM-1, copeptin, and normetanephrine, the study may be underpowered for these outcomes. Therefore, conclusions such as “heat acclimation did not alter renal stress markers” should be interpreted more cautiously. The absence of statistical significance does not necessarily indicate the absence of a physiological effect.

We acknowledge this important concern by the reviewer – we have addressed the reasons why in reviewer 1 comments. Further to this we have tempered our conclusions to show the caution we have when interpreting these results and emphasise this in our limitations section.

2. Lack of direct renal function measurements
The manuscript discusses renal stress and AKI risk extensively; however, no direct indices of renal function (e.g., serum creatinine, cystatin C, eGFR, urinary albumin, or creatinine clearance) were measured. While NGAL and KIM-1 are useful early tubular stress biomarkers, they do not alone establish kidney dysfunction or AKI risk. The discussion and conclusion should therefore avoid overinterpretation regarding “renal protection” or unchanged “kidney injury risk.”

We thank the reviewer for this important comment. We have adjusted statements around it ‘does not’ to it ‘may not’ and have been more specific around markers of early tubular stress and not more broadly to renal protection or kidney function. This study has several limitations that should be considered when interpreting the findings. We did not include direct measures of kidney function (e.g. serum creatinine or glomerular filtration rate). While this was intentional to focus on emerging early tubular injury markers (NGAL and KIM-1) and neuroendocrine indices (copeptin, NMET), it limits inferences about acute changes in filtration and the functional significance of any biomarker shifts. Second, kidney injury markers were assessed only in blood. Although circulating concentrations of NGAL and KIM-1 offer practical sampling during repeated exercise-heat exposures, they are not kidney-exclusive and may be influenced by extra renal sources and systemic inflammation. Future research should include a concurrent urinary biomarker panel to better understand tubular site specificity

3. Exercise workload for each intervention
The description of the TEMP intervention states that exercise was performed at a “matched workload”; however, it is unclear how workload matching between HOT and TEMP conditions was achieved. In the HOT group, exercise intensity appears to have been adjusted dynamically to maintain core temperature at ≥38.5°C, including periods of passive or reduced-intensity exercise. Please clarify whether workload matching refers to external workload, exercise duration, total work completed, metabolic heat production, or relative exercise intensity. In addition, please report whether the total work performed differed between groups across the five intervention sessions. This information is important for interpreting whether the observed adaptations were attributable to environmental heat exposure rather than differences in exercise stimulus alone. Also, please clarify how core temperature was measured during the intervention sessions.

Thank you for this important observation, we have now included the following statement into the results. ‘There were no significant differences (*p* > 0.05) in exercise duration or relative exercise PO, suggesting that a similar training stress was induced between groups. Although, the HOT group exercised at a higher percentage of their HR_max_ compared to TEMP (79 vs. 71%, *p* = 0.030, respectively), indicating the magnitude of cardiovascular strain was different between groups.’

4. Interpretation of null findings
Several mechanistic interpretations are stronger than the data support. For example, statements implying preserved renal perfusion or unchanged tubular injury risk based on unchanged copeptin and normetanephrine responses should be tempered, as renal blood flow/perfusion was not directly measured.

Once again, thank you for this observation. Based off the same limitations from reviewer 1 we have tempered our statements around changes in renal blood flow.

5. Timing of biomarker collection
Blood biomarkers were collected immediately post-exercise only. Some renal injury biomarkers may exhibit delayed peak responses after exercise-heat stress. This limitation should be discussed more explicitly.

The authors acknowledge this limitation and have included the following in the limitation section ‘The blood biomarker analysis was limited to a single time-point after the heat stress test, which may not have coincided with the peak response, specifically NGAL and KIM-1. However, immediately after exercise was chosen to capture peak physiological perturbations associated with heat stress, reflecting peak responses in copeptin, osmolality and NMET which rise rapidly with haemodynamic and osmotic stress on the kidney.

Minor Comments
1. Please clarify participant sex distribution in Table 1 and/or Methods.

All participants were male, this has been reflected in the abstract and methods.

2. Please clarify whether dietary intake, caffeine, alcohol, and hydration were standardized prior to heat stress testing.

Participants were asked to maintain their normal diet, and avoid caffeine intake 12 hours prior, alcohol 24 hours prior and to arrive euhydrated as confirmed by hydration status measurement. See design of study section.

3. Figure 1 would be strengthened by displaying both pre- and post-exercise biomarker values within each heat stress test.

We thank the reviewer for this comment, but have decided that the current figure is sufficient for the results interpretation, especially with no significant change in any biomarker.

4. The “New & Noteworthy” section slightly overstates the renal conclusions. Consider replacing: “did not reduce renal biomarker responses” with: “did not clearly attenuate renal biomarker responses.”

This comment has been integrated into the abstract and conclusion statements, but the new and noteworthy section has been deleted as not a requirement for PLOS ONE submission.
