# Supplementary material for: Five days of heat acclimation improves cardiovascular and thermoregulatory responses without altering renal stress biomarkers in endurance athletes
Source: PLoS One. 2026 Aug 11;21(8):e0345346. doi: 10.1371/journal.pone.0345346 (PMC13460581; doi:10.1371/journal.pone.0345346)
Supplement: Supplementary file 2 [file pone.0345346.s003.docx]

*Reviewer #1: Thanks for addressing my comments. I hope you'll agree that the manuscript has improved, and conclusions drawn are more aligned with the data presented.*

Agreed. The authors would like to thank you for a thorough and fair review of our manuscript.

*Reviewer #2: I would like to thank the authors for their careful revision of the manuscript and their detailed responses to the reviewers’ comments. The revised version is substantially improved compared with the original submission. In particular, the clarification of the statistical analyses, the improved description of the statistical procedures, the inclusion of effect sizes and confidence intervals, the expanded limitations section, and the more cautious interpretation of the findings have strengthened the scientific rigor and transparency of the study. Most of my previous comments have been adequately addressed. However, one important concern remains regarding the matching of exercise intensity between the intervention groups.*

*Major Comment
1. Exercise intensity matching remains insufficiently justified.
The response regarding exercise intensity does not fully resolve my previous concern. The authors state that exercise duration and external workload were matched between groups using a relative power output of 2.5 W·kg⁻¹. However, matching external workload relative to body mass does not necessarily indicate equivalent physiological exercise intensity. Importantly, maximal ramp minute power (MRMP) was significantly higher in the HOT group than in the TEMP group at baseline. Furthermore, the authors report that the HOT group exercised at a significantly higher percentage of maximal heart rate during the intervention period (79% vs. 71% HRmax). These findings suggest that the physiological training stimulus may not have been equivalent between groups. Consequently, it remains difficult to determine whether the observed adaptations were attributable solely to heat acclimation or were influenced by differences in exercise intensity and cardiovascular strain between groups. I therefore recommend that the authors:
1. Clarify how exercise intensity was adjusted and monitored to achieve and maintain the target core temperature of 38.5 °C in the HOT group. In the main text, “Exercise intensity was set at 2.5 W·kg⁻¹ to raise core temperature to 38.5 °C within ~30-minutes, followed by passive or reduced-intensity exercise to maintain target temperature”(page 6). The current description indicates that exercise intensity was not constant, but passive or reduced-intensity exercise was followed after reaching the target temperature. Therefore, the actual exercise stimulus during the 90-minute HA sessions remains unclear.
2. Clarify how exercise intensity was controlled and matched in the TEMP group throughout the intervention period.
3. Report more comprehensively the physiological exercise intensity experienced by each group during the intervention (e.g., %HRmax, %MRMP, %V̇O₂max, average power output, total work performed, or other relevant indices).
4. Expand the Discussion and Limitations sections to acknowledge that differences in baseline aerobic fitness and cardiovascular strain during the intervention may have influenced the observed adaptations.
Without additional clarification, the interpretation that the observed responses are attributable exclusively to heat acclimation remains insufficiently supported.*

We thank the reviewer for this thoughtful comment and agree that further clarification of the exercise prescription and intervention methodology is warranted.

To address this concern, we have revised the Methods section to more clearly describe how exercise intensity was prescribed and adjusted throughout the intervention period. Specifically, participants in both groups initially exercised at 2.5 W·kg⁻¹. Thereafter, exercise intensity was self-selected for the remainder of the session. In the HOT group, exercise intensity was adjusted as required to achieve and maintain a rectal temperature ≥38.5 °C, with passive recovery permitted when rectal temperature exceeded 39.3 °C. In contrast, the TEMP group followed the same general exercise structure in temperate conditions but without a prescribed core temperature target.

Accordingly, we have removed the term "matched" when describing exercise intensity within the Abstract and Methods, as this may have implied that physiological responses were expected to be equivalent between groups. Rather, the intervention was designed to maintain a comparable exercise prescription while manipulating thermal load through the controlled-hyperthermia methodology.

We acknowledge the reviewer's observation that participants in the HOT group exercised at a higher percentage of maximal heart rate during the intervention period. However, this finding is consistent with the expected cardiovascular demands associated with exercising in a hot environment and is inherent to controlled-hyperthermia heat-acclimation protocols. Importantly, exercise duration and average relative power output were not different between groups (as cited in the results), indicating a comparable overall training volume despite the greater cardiovascular and thermal strain experienced in HOT. Therefore, while cardiovascular responses differed between conditions, these differences are reflective of the intended thermal stimulus rather than differences in prescribed exercise volume.

We hope these revisions provide greater clarity regarding the intervention methodology and support the interpretation of the observed adaptations.
