# Supplementary material for: Five days of heat acclimation improves cardiovascular and thermoregulatory responses without altering renal stress biomarkers in endurance athletes
Source: PLoS One. 2026 Aug 11;21(8):e0345346. doi: 10.1371/journal.pone.0345346 (PMC13460581; doi:10.1371/journal.pone.0345346)
Supplement: Supplementary file 3 [file pone.0345346.s004.docx]

Reviewer #2: I would like to thank the authors for revising the manuscript and for their detailed responses to the reviewers’ comments. I have only one remaining minor comment.
The intervention protocol is now much clearer. However, I would appreciate a brief additional description of how core temperature was monitored during each heat acclimation session.
The manuscript states that exercise intensity was initially set at 2.5 W·kg⁻¹ to increase core temperature to ≥38.5°C, after which exercise intensity was self-selected to maintain this target temperature, with passive recovery permitted when core temperature exceeded 39.3°C. While this adequately describes the intervention strategy, it remains unclear how core temperature was monitored in practice during the intervention sessions. Providing these methodological details would further improve the reproducibility of the heat acclimation protocol.

Once again, we would like to sincerely thank the reviewers for their feedback. We have addressed this comment and specified the measurement of rectal body temperature as our method to obtain core temperature. I have amended this in our updated manuscript.
