# Supplementary material for: Five days of heat acclimation improves cardiovascular and thermoregulatory responses without altering renal stress biomarkers in endurance athletes
Source: PLoS One. 2026 Aug 11;21(8):e0345346. doi: 10.1371/journal.pone.0345346 (PMC13460581; doi:10.1371/journal.pone.0345346)
Supplement: Peer review history

# Peer review: Decision Letter 0

**DOI:** 10.1371/journal.pone.0345346.r001

**Authors:** Hidenori Otani

27 May 2026

Dear Dr. Snape,

Thank you for submitting your manuscript to PLOS ONE. After careful consideration, we feel that it has merit but does not fully meet PLOS ONE’s publication criteria as it currently stands. Therefore, we invite you to submit a revised version of the manuscript that addresses the points raised during the review process.

Please submit your revised manuscript by Jul 11 2026 11:59PM. If you will need more time than this to complete your revisions, please reply to this message or contact the journal office at plosone@plos.org. When you're ready to submit your revision, log on to https://www.editorialmanager.com/pone/ and select the 'Submissions Needing Revision' folder to locate your manuscript file.

- A letter that responds to each point raised by the academic editor and reviewer(s). You should upload this letter as a separate file labeled 'Response to Reviewers'.
- A marked-up copy of your manuscript that highlights changes made to the original version. You should upload this as a separate file labeled 'Revised Manuscript with Track Changes'.
- An unmarked version of your revised paper without tracked changes. You should upload this as a separate file labeled 'Manuscript'.

If you would like to make changes to your financial disclosure, please include your updated statement in your cover letter. Guidelines for resubmitting your figure files are available below the reviewer comments at the end of this letter.

If applicable, we recommend that you deposit your laboratory protocols in protocols.io to enhance the reproducibility of your results. Protocols.io assigns your protocol its own identifier (DOI) so that it can be cited independently in the future. For instructions see: https://journals.plos.org/plosone/s/submission-guidelines#loc-laboratory-protocols. Additionally, PLOS ONE offers an option for publishing peer-reviewed Lab Protocol articles, which describe protocols hosted on protocols.io. Read more information on sharing protocols at https://plos.org/protocols?utm_medium=editorial-email&utm_source=authorletters&utm_campaign=protocols.

As the corresponding author, your ORCID iD is verified in the submission system and will appear in the published article. PLOS supports the use of ORCID, and we encourage all coauthors to register for an ORCID iD and use it as well. Please encourage your coauthors to verify their ORCID iD within the submission system before final acceptance, as unverified ORCID iDs will not appear in the published article. *Only*  the individual author can complete the verification step; PLOS staff *cannot*  verify ORCID iDs on behalf of authors.

We look forward to receiving your revised manuscript.

Kind regards,

Hidenori Otani, Ph.D.

Academic Editor

PLOS One

Journal Requirements:

When submitting your revision, we need you to address these additional requirements.

1. Please ensure that your manuscript meets PLOS ONE's style requirements, including those for file naming. The PLOS ONE style templates can be found at

https://journals.plos.org/plosone/s/file?id=wjVg/PLOSOne_formatting_sample_main_body.pdf and https://journals.plos.org/plosone/s/file?id=ba62/PLOSOne_formatting_sample_title_authors_affiliations.pdf

2. In the online submission form, you indicated that the dataset analysed during the current study may be requested upon an informal inquiry addressed to the corresponding author.

All PLOS journals now require all data underlying the findings described in their manuscript to be freely available to other researchers, either 1. In a public repository, 2. Within the manuscript itself, or 3. Uploaded as supplementary information.

This policy applies to all data except where public deposition would breach compliance with the protocol approved by your research ethics board. If your data cannot be made publicly available for ethical or legal reasons (e.g., public availability would compromise patient privacy), please explain your reasons on resubmission and your exemption request will be escalated for approval.

3. If the reviewer comments include a recommendation to cite specific previously published works, please review and evaluate these publications to determine whether they are relevant and should be cited. There is no requirement to cite these works unless the editor has indicated otherwise.

[Note: HTML markup is below. Please do not edit.]

Reviewers' comments:

Reviewer's Responses to Questions

**Comments to the Author**

1. Is the manuscript technically sound, and do the data support the conclusions?

Reviewer #1: Yes

Reviewer #2: Yes

**********

2. Has the statistical analysis been performed appropriately and rigorously?

Reviewer #1: Yes

Reviewer #2: Yes

**********

3. Have the authors made all data underlying the findings in their manuscript fully available?

The PLOS Data policy

Reviewer #1: Yes

Reviewer #2: Yes

**********

4. Is the manuscript presented in an intelligible fashion and written in standard English?

Reviewer #1: Yes

Reviewer #2: Yes

**********

Reviewer #1: Snape et al. investigate the effects of a 5-day heat acclimation (HA) protocol on cardiovascular and thermoregulatory responses, alongside renal stress biomarkers, in endurance-trained individuals. The study appears to be methodologically sound overall and addresses a relevant applied physiology question. However, I have several concerns regarding the framing of the hypotheses and the interpretation of the renal outcomes. In particular, the rationale underpinning the renal hypotheses is not fully convincing, and the null findings are, at times, overstated. The comments below are intended to help improve the clarity, balance, and physiological grounding of the manuscript.

Minor Comments

Introduction

“Improved plasma volume and sympathetic modulation following HA may preserve renal blood flow…”

Please provide a citation to support this statement. It is not clear that HA would preferentially preserve renal blood flow during exercise heat stress. If anything, the combination of heat stress and exercise is typically associated with renal vasoconstriction and reduced renal perfusion. The physiological basis for this statement requires further justification.

“These cardiovascular adaptations could offset the renal hypoperfusion…”

At present, the Introduction does not convincingly support the idea that HA could meaningfully offset reductions in renal blood flow during exercise in the heat. This point would benefit from either stronger supporting evidence or more cautious phrasing.

“there influence…”

Typographical error: should read “their influence”.

“the kidneys exhibited an increased sensitivity to hyperthermia…”

This statement is too broad. Please clarify what is meant by “increased sensitivity” and specify the physiological evidence supporting this claim.

The hypothesis that HA would attenuate renal tubular injury biomarkers appears insufficiently justified. You cite Connor et al., who used a longer HA protocol and reported no effect on NGAL, yet still hypothesise an attenuation with a shorter intervention. Please provide a clearer mechanistic or empirical rationale for this expectation.

Methods

Heat stress test (HST):

You refer to gastrointestinal temperature (Tgi) but later mention rectal temperature (Tre). Please clarify which measure was used and ensure consistency throughout.

Blood sampling timing:

Please provide more detail on the timing of blood sampling during the HSTs, particularly the “post” sample. Was this obtained immediately post-exercise or after a delay? This is important for interpretation of biomarker responses.

TEMP intervention description:

The TEMP condition is described as being performed at a “matched workload,” but it is unclear what this corresponds to (2.5 W/kg?). Given that the HOT condition is prescribed relative to core temperature, additional clarification is needed to allow comparison between conditions.

Discussion

“reductions in neurohumoral markers… may have helped keep blood vessels in the kidneys more open…”

This statement would benefit from supporting evidence or a clearer mechanistic explanation. At present, the link between changes in these markers and renal vascular conductance during exercise heat stress is not well established.

Heat training and renal stress (Rønnestad et al., 2021)

This section appears somewhat inconsistent with the earlier hypothesis that HA may attenuate renal stress. If HA is proposed to be protective, the suggestion that repeated heat exposure could increase renal risk requires clearer reconciliation.

“no change observed… may reflect an underpowered design”

This statement is not fully convincing in its current form. Given that renal biomarkers represent a key novel aspect of the study, it would have been appropriate to base sample size calculations on expected changes in these variables. As prior literature (including studies cited by the authors) has reported similar null findings, this possibility should have been considered more explicitly in the study design and discussed more transparently here.

Conclusion

“…longer HA protocols may be required… which could confer greater protection against renal injury…”

Based on the current data, this statement appears somewhat speculative. While it is reasonable to suggest that longer protocols may induce a more complete heat-adapted phenotype, the implication that this would translate to renal protection is not clearly supported by the findings presented. Consider tempering this statement or more clearly framing it as a hypothesis for future work.

Figures

Consider including the outcomes of the two-way ANOVAs directly on the figures (e.g., main effects and interactions). This would improve readability and reduce the need to cross-reference figure captions.

Reviewer #2: This manuscript investigates whether five days of short-term isothermic heat acclimation influences renal stress biomarkers and neuroendocrine responses during exercise-heat stress in endurance-trained athletes. The topic is timely and relevant given the increasing concern regarding exercise-associated kidney stress during training and competition in hot environments. The inclusion of renal tubular biomarkers (NGAL and KIM-1), alongside copeptin and normetanephrine, is a notable strength that increases the study's novelty. The study design is generally appropriate, and the authors successfully demonstrated classical heat acclimation adaptations, including reduced resting heart rate, plasma volume expansion, and increased sweat loss. Several important methodological and interpretative concerns should be addressed before the manuscript can be considered for publication.

Major Comments

1. Statistical power for biomarker outcomes

The sample size calculation appears to have been based primarily on thermoregulatory variables rather than renal biomarkers. Given the substantial inter-individual variability in responses to NGAL, KIM-1, copeptin, and normetanephrine, the study may be underpowered for these outcomes. Therefore, conclusions such as “heat acclimation did not alter renal stress markers” should be interpreted more cautiously. The absence of statistical significance does not necessarily indicate the absence of a physiological effect.

2. Lack of direct renal function measurements

The manuscript discusses renal stress and AKI risk extensively; however, no direct indices of renal function (e.g., serum creatinine, cystatin C, eGFR, urinary albumin, or creatinine clearance) were measured. While NGAL and KIM-1 are useful early tubular stress biomarkers, they do not alone establish kidney dysfunction or AKI risk. The discussion and conclusion should therefore avoid overinterpretation regarding “renal protection” or unchanged “kidney injury risk.”

3. Exercise workload for each intervention

The description of the TEMP intervention states that exercise was performed at a “matched workload”; however, it is unclear how workload matching between HOT and TEMP conditions was achieved. In the HOT group, exercise intensity appears to have been adjusted dynamically to maintain core temperature at ≥38.5°C, including periods of passive or reduced-intensity exercise. Please clarify whether workload matching refers to external workload, exercise duration, total work completed, metabolic heat production, or relative exercise intensity. In addition, please report whether the total work performed differed between groups across the five intervention sessions. This information is important for interpreting whether the observed adaptations were attributable to environmental heat exposure rather than differences in exercise stimulus alone. Also, please clarify how core temperature was measured during the intervention sessions.

4. Interpretation of null findings

Several mechanistic interpretations are stronger than the data support. For example, statements implying preserved renal perfusion or unchanged tubular injury risk based on unchanged copeptin and normetanephrine responses should be tempered, as renal blood flow/perfusion was not directly measured.

5. Timing of biomarker collection

Blood biomarkers were collected immediately post-exercise only. Some renal injury biomarkers may exhibit delayed peak responses after exercise-heat stress. This limitation should be discussed more explicitly.

Minor Comments

1. Please clarify participant sex distribution in Table 1 and/or Methods.

2. Please clarify whether dietary intake, caffeine, alcohol, and hydration were standardized prior to heat stress testing.

3. Figure 1 would be strengthened by displaying both pre- and post-exercise biomarker values within each heat stress test.

4. The “New & Noteworthy” section slightly overstates the renal conclusions. Consider replacing: “did not reduce renal biomarker responses” with: “did not clearly attenuate renal biomarker responses.”

**********

what does this mean?). If published, this will include your full peer review and any attached files.

If you choose “no”, your identity will remain anonymous but your review may still be made public.

**Do you want your identity to be public for this peer review?**  For information about this choice, including consent withdrawal, please see our Privacy Policy

Reviewer #1: No

Reviewer #2: **Yes:** Kazunobu Okazaki

**********

[NOTE: If reviewer comments were submitted as an attachment file, they will be attached to this email and accessible via the submission site. Please log into your account, locate the manuscript record, and check for the action link "View Attachments". If this link does not appear, there are no attachment files.]

To ensure your figures meet our technical requirements, please review our figure guidelines: https://journals.plos.org/plosone/s/figures

You may also use PLOS’s free figure tool, NAAS, to help you prepare publication quality figures: https://journals.plos.org/plosone/s/figures#loc-tools-for-figure-preparation.

NAAS will assess whether your figures meet our technical requirements by comparing each figure against our figure specifications.

# Author comment: Author response to Decision Letter 1

**DOI:** 10.1371/journal.pone.0345346.r002

4 Jun 2026

We thank the editor for being considered for publication in this journal and the reviewers for their constructive feedback which has been considered and implemented were applicable. We know believe this manuscript has been improved and addresses major and minor comments requested. Please see my 'response to reviewers' document for further information.

Attachment Submitted filename: Response_to_reviewers.docx

# Peer review: Decision Letter 1

**DOI:** 10.1371/journal.pone.0345346.r003

**Authors:** Hidenori Otani

17 Jun 2026

Dear Dr. Snape,

Thank you for submitting your manuscript to PLOS ONE. After careful consideration, we feel that it has merit but does not fully meet PLOS ONE’s publication criteria as it currently stands. Therefore, we invite you to submit a revised version of the manuscript that addresses the points raised during the review process.

Please submit your revised manuscript by Aug 01 2026 11:59PM. If you will need more time than this to complete your revisions, please reply to this message or contact the journal office at plosone@plos.org. When you're ready to submit your revision, log on to https://www.editorialmanager.com/pone/ and select the 'Submissions Needing Revision' folder to locate your manuscript file.

- A letter that responds to each point raised by the academic editor and reviewer(s). You should upload this letter as a separate file labeled 'Response to Reviewers'.
- A marked-up copy of your manuscript that highlights changes made to the original version. You should upload this as a separate file labeled 'Revised Manuscript with Track Changes'.
- An unmarked version of your revised paper without tracked changes. You should upload this as a separate file labeled 'Manuscript'.

If you would like to make changes to your financial disclosure, please include your updated statement in your cover letter. Guidelines for resubmitting your figure files are available below the reviewer comments at the end of this letter.

If applicable, we recommend that you deposit your laboratory protocols in protocols.io to enhance the reproducibility of your results. Protocols.io assigns your protocol its own identifier (DOI) so that it can be cited independently in the future. For instructions see: https://journals.plos.org/plosone/s/submission-guidelines#loc-laboratory-protocols. Additionally, PLOS ONE offers an option for publishing peer-reviewed Lab Protocol articles, which describe protocols hosted on protocols.io. Read more information on sharing protocols at https://plos.org/protocols?utm_medium=editorial-email&utm_source=authorletters&utm_campaign=protocols.

As the corresponding author, your ORCID iD is verified in the submission system and will appear in the published article. PLOS supports the use of ORCID, and we encourage all coauthors to register for an ORCID iD and use it as well. Please encourage your coauthors to verify their ORCID iD within the submission system before final acceptance, as unverified ORCID iDs will not appear in the published article. *Only*  the individual author can complete the verification step; PLOS staff *cannot*  verify ORCID iDs on behalf of authors.

We look forward to receiving your revised manuscript.

Kind regards,

Hidenori Otani, Ph.D.

Academic Editor

PLOS One

Journal Requirements:

1. If the reviewer comments include a recommendation to cite specific previously published works, please review and evaluate these publications to determine whether they are relevant and should be cited. There is no requirement to cite these works unless the editor has indicated otherwise.

2. Please review your reference list to ensure that it is complete and correct. If you have cited papers that have been retracted, please include the rationale for doing so in the manuscript text, or remove these references and replace them with relevant current references. Any changes to the reference list should be mentioned in the rebuttal letter that accompanies your revised manuscript. If you need to cite a retracted article, indicate the article’s retracted status in the References list and also include a citation and full reference for the retraction notice.

[Note: HTML markup is below. Please do not edit.]

Reviewers' comments:

Reviewer's Responses to Questions

**Comments to the Author**

Reviewer #1: All comments have been addressed

Reviewer #2: All comments have been addressed

**********

2. Is the manuscript technically sound, and do the data support the conclusions?

Reviewer #1: Yes

Reviewer #2: Yes

**********

3. Has the statistical analysis been performed appropriately and rigorously?

Reviewer #1: Yes

Reviewer #2: Yes

**********

4. Have the authors made all data underlying the findings in their manuscript fully available?

The PLOS Data policy

Reviewer #1: Yes

Reviewer #2: Yes

**********

5. Is the manuscript presented in an intelligible fashion and written in standard English?

Reviewer #1: Yes

Reviewer #2: Yes

**********

Reviewer #1: Thanks for addressing my comments. I hope you'll agree that the manuscript has improved, and conclusions drawn are more aligned with the data presented.

Reviewer #2: I would like to thank the authors for their careful revision of the manuscript and their detailed responses to the reviewers’ comments. The revised version is substantially improved compared with the original submission. In particular, the clarification of the statistical analyses, the improved description of the statistical procedures, the inclusion of effect sizes and confidence intervals, the expanded limitations section, and the more cautious interpretation of the findings have strengthened the scientific rigor and transparency of the study. Most of my previous comments have been adequately addressed. However, one important concern remains regarding the matching of exercise intensity between the intervention groups.

Major Comment

1. Exercise intensity matching remains insufficiently justified.

The response regarding exercise intensity does not fully resolve my previous concern. The authors state that exercise duration and external workload were matched between groups using a relative power output of 2.5 W·kg⁻¹. However, matching external workload relative to body mass does not necessarily indicate equivalent physiological exercise intensity. Importantly, maximal ramp minute power (MRMP) was significantly higher in the HOT group than in the TEMP group at baseline. Furthermore, the authors report that the HOT group exercised at a significantly higher percentage of maximal heart rate during the intervention period (79% vs. 71% HRmax). These findings suggest that the physiological training stimulus may not have been equivalent between groups. Consequently, it remains difficult to determine whether the observed adaptations were attributable solely to heat acclimation or were influenced by differences in exercise intensity and cardiovascular strain between groups. I therefore recommend that the authors:

1. Clarify how exercise intensity was adjusted and monitored to achieve and maintain the target core temperature of 38.5 °C in the HOT group. In the main text, “Exercise intensity was set at 2.5 W·kg⁻¹ to raise core temperature to 38.5 °C within ~30-minutes, followed by passive or reduced-intensity exercise to maintain target temperature”(page 6). The current description indicates that exercise intensity was not constant, but passive or reduced-intensity exercise was followed after reaching the target temperature. Therefore, the actual exercise stimulus during the 90-minute HA sessions remains unclear.

2. Clarify how exercise intensity was controlled and matched in the TEMP group throughout the intervention period.

3. Report more comprehensively the physiological exercise intensity experienced by each group during the intervention (e.g., %HRmax, %MRMP, %V̇O₂max, average power output, total work performed, or other relevant indices).

4. Expand the Discussion and Limitations sections to acknowledge that differences in baseline aerobic fitness and cardiovascular strain during the intervention may have influenced the observed adaptations.

Without additional clarification, the interpretation that the observed responses are attributable exclusively to heat acclimation remains insufficiently supported.

**********

what does this mean?). If published, this will include your full peer review and any attached files.

If you choose “no”, your identity will remain anonymous but your review may still be made public.

**Do you want your identity to be public for this peer review?**  For information about this choice, including consent withdrawal, please see our Privacy Policy

Reviewer #1: No

Reviewer #2: **Yes:** Kazunobu Okazaki

**********

[NOTE: If reviewer comments were submitted as an attachment file, they will be attached to this email and accessible via the submission site. Please log into your account, locate the manuscript record, and check for the action link "View Attachments". If this link does not appear, there are no attachment files.]

To ensure your figures meet our technical requirements, please review our figure guidelines: https://journals.plos.org/plosone/s/figures

You may also use PLOS’s free figure tool, NAAS, to help you prepare publication quality figures: https://journals.plos.org/plosone/s/figures#loc-tools-for-figure-preparation.

NAAS will assess whether your figures meet our technical requirements by comparing each figure against our figure specifications.

# Author comment: Author response to Decision Letter 2

**DOI:** 10.1371/journal.pone.0345346.r004

20 Jul 2026

We thank the reviewer for this thoughtful comment and agree that further clarification of the exercise prescription and intervention methodology is warranted.

To address this concern, we have revised the Methods section to more clearly describe how exercise intensity was prescribed and adjusted throughout the intervention period. Specifically, participants in both groups initially exercised at 2.5 W·kg⁻¹. Thereafter, exercise intensity was self-selected for the remainder of the session. In the HOT group, exercise intensity was adjusted as required to achieve and maintain a rectal temperature ≥38.5 °C, with passive recovery permitted when rectal temperature exceeded 39.3 °C. In contrast, the TEMP group followed the same general exercise structure in temperate conditions but without a prescribed core temperature target.

Accordingly, we have removed the term "matched" when describing exercise intensity within the Abstract and Methods, as this may have implied that physiological responses were expected to be equivalent between groups. Rather, the intervention was designed to maintain a comparable exercise prescription while manipulating thermal load through the controlled-hyperthermia methodology.

We acknowledge the reviewer's observation that participants in the HOT group exercised at a higher percentage of maximal heart rate during the intervention period. However, this finding is consistent with the expected cardiovascular demands associated with exercising in a hot environment and is inherent to controlled-hyperthermia heat-acclimation protocols. Importantly, exercise duration and average relative power output were not different between groups (as cited in the results), indicating a comparable overall training volume despite the greater cardiovascular and thermal strain experienced in HOT. Therefore, while cardiovascular responses differed between conditions, these differences are reflective of the intended thermal stimulus rather than differences in prescribed exercise volume.

We hope these revisions provide greater clarity regarding the intervention methodology and support the interpretation of the observed adaptations.

Attachment Submitted filename: Response to reviewers_V2.docx

# Peer review: Decision Letter 2

**DOI:** 10.1371/journal.pone.0345346.r005

**Authors:** Hidenori Otani

22 Jul 2026

Dear Dr. Snape,

Thank you for submitting your manuscript to PLOS ONE. After careful consideration, we feel that it has merit but does not fully meet PLOS ONE’s publication criteria as it currently stands. Therefore, we invite you to submit a revised version of the manuscript that addresses the points raised during the review process.

Please submit your revised manuscript by Sep 05 2026 11:59PM. If you will need more time than this to complete your revisions, please reply to this message or contact the journal office at plosone@plos.org. When you're ready to submit your revision, log on to https://www.editorialmanager.com/pone/ and select the 'Submissions Needing Revision' folder to locate your manuscript file.

- A letter that responds to each point raised by the academic editor and reviewer(s). You should upload this letter as a separate file labeled 'Response to Reviewers'.
- A marked-up copy of your manuscript that highlights changes made to the original version. You should upload this as a separate file labeled 'Revised Manuscript with Track Changes'.
- An unmarked version of your revised paper without tracked changes. You should upload this as a separate file labeled 'Manuscript'.

If you would like to make changes to your financial disclosure, please include your updated statement in your cover letter. Guidelines for resubmitting your figure files are available below the reviewer comments at the end of this letter.

If applicable, we recommend that you deposit your laboratory protocols in protocols.io to enhance the reproducibility of your results. Protocols.io assigns your protocol its own identifier (DOI) so that it can be cited independently in the future. For instructions see: https://journals.plos.org/plosone/s/submission-guidelines#loc-laboratory-protocols. Additionally, PLOS ONE offers an option for publishing peer-reviewed Lab Protocol articles, which describe protocols hosted on protocols.io. Read more information on sharing protocols at https://plos.org/protocols?utm_medium=editorial-email&utm_source=authorletters&utm_campaign=protocols.

As the corresponding author, your ORCID iD is verified in the submission system and will appear in the published article. PLOS supports the use of ORCID, and we encourage all coauthors to register for an ORCID iD and use it as well. Please encourage your coauthors to verify their ORCID iD within the submission system before final acceptance, as unverified ORCID iDs will not appear in the published article. *Only*  the individual author can complete the verification step; PLOS staff *cannot*  verify ORCID iDs on behalf of authors.

We look forward to receiving your revised manuscript.

Kind regards,

Hidenori Otani, Ph.D.

Academic Editor

PLOS One

Journal Requirements:

If the reviewer comments include a recommendation to cite specific previously published works, please review and evaluate these publications to determine whether they are relevant and should be cited. There is no requirement to cite these works unless the editor has indicated otherwise.

Please review your reference list to ensure that it is complete and correct. If you have cited papers that have been retracted, please include the rationale for doing so in the manuscript text, or remove these references and replace them with relevant current references. Any changes to the reference list should be mentioned in the rebuttal letter that accompanies your revised manuscript. If you need to cite a retracted article, indicate the article’s retracted status in the References list and also include a citation and full reference for the retraction notice.

[Note: HTML markup is below. Please do not edit.]

Reviewers' comments:

Reviewer's Responses to Questions

**Comments to the Author**

Reviewer #2: All comments have been addressed

**********

2. Is the manuscript technically sound, and do the data support the conclusions?

Reviewer #2: Yes

**********

3. Has the statistical analysis been performed appropriately and rigorously?

Reviewer #2: Yes

**********

4. Have the authors made all data underlying the findings in their manuscript fully available?

The PLOS Data policy

Reviewer #2: Yes

**********

5. Is the manuscript presented in an intelligible fashion and written in standard English?

Reviewer #2: Yes

**********

Reviewer #2: I would like to thank the authors for revising the manuscript and for their detailed responses to the reviewers’ comments. I have only one remaining minor comment.

The intervention protocol is now much clearer. However, I would appreciate a brief additional description of how core temperature was monitored during each heat acclimation session.

The manuscript states that exercise intensity was initially set at 2.5 W·kg⁻¹ to increase core temperature to ≥38.5°C, after which exercise intensity was self-selected to maintain this target temperature, with passive recovery permitted when core temperature exceeded 39.3°C. While this adequately describes the intervention strategy, it remains unclear how core temperature was monitored in practice during the intervention sessions. Providing these methodological details would further improve the reproducibility of the heat acclimation protocol.

**********

what does this mean?). If published, this will include your full peer review and any attached files.

If you choose “no”, your identity will remain anonymous but your review may still be made public.

**Do you want your identity to be public for this peer review?**  For information about this choice, including consent withdrawal, please see our Privacy Policy

Reviewer #2: **Yes:** Kazunobu Okazaki

**********

[NOTE: If reviewer comments were submitted as an attachment file, they will be attached to this email and accessible via the submission site. Please log into your account, locate the manuscript record, and check for the action link "View Attachments". If this link does not appear, there are no attachment files.]

To ensure your figures meet our technical requirements, please review our figure guidelines: https://journals.plos.org/plosone/s/figures

You may also use PLOS’s free figure tool, NAAS, to help you prepare publication quality figures: https://journals.plos.org/plosone/s/figures#loc-tools-for-figure-preparation.

NAAS will assess whether your figures meet our technical requirements by comparing each figure against our figure specifications.

# Author comment: Author response to Decision Letter 3

**DOI:** 10.1371/journal.pone.0345346.r006

24 Jul 2026

I have updated the manuscript to specify rectal temperature as our method off measuring core temperature. We thank the reviewer for this important observation and the chance to correct this.

Attachment Submitted filename: Response to reviewers_V3.docx

# Peer review: Decision Letter 3

**DOI:** 10.1371/journal.pone.0345346.r007

**Authors:** Hidenori Otani

28 Jul 2026

Five days of heat acclimation improves cardiovascular and thermoregulatory responses without altering renal stress biomarkers in endurance athletes

PONE-D-26-10537R3

Dear Dr. Snape,

We’re pleased to inform you that your manuscript has been judged scientifically suitable for publication and will be formally accepted for publication once it meets all outstanding technical requirements.

Within one week, you’ll receive an e-mail detailing the required amendments. When these have been addressed, you’ll receive a formal acceptance letter and your manuscript will be scheduled for publication.

An invoice will be generated when your article is formally accepted. Please note, if your institution has a publishing partnership with PLOS and your article meets the relevant criteria, all or part of your publication costs will be covered. Please make sure your user information is up-to-date by logging into Editorial Manager at Editorial Manager® and clicking the ‘Update My Information' link at the top of the page. For questions related to billing, please contact billing support.

If your institution or institutions have a press office, please notify them about your upcoming paper to help maximize its impact. If they’ll be preparing press materials, please inform our press team as soon as possible -- no later than 48 hours after receiving the formal acceptance. Your manuscript will remain under strict press embargo until 2 pm Eastern Time on the date of publication. For more information, please contact onepress@plos.org.

Kind regards,

Hidenori Otani, Ph.D.

Academic Editor

PLOS One

Additional Editor Comments (optional):

Reviewers' comments:

Reviewer's Responses to Questions

**Comments to the Author**

Reviewer #2: All comments have been addressed

**********

2. Is the manuscript technically sound, and do the data support the conclusions?

Reviewer #2: Yes

**********

3. Has the statistical analysis been performed appropriately and rigorously?

Reviewer #2: Yes

**********

4. Have the authors made all data underlying the findings in their manuscript fully available?

The PLOS Data policy

Reviewer #2: Yes

**********

5. Is the manuscript presented in an intelligible fashion and written in standard English?

Reviewer #2: Yes

**********

Reviewer #2: I would like to thank the authors for their careful revision of the manuscript and their detailed responses to my previous comments. I have no further comments.

**********

what does this mean?). If published, this will include your full peer review and any attached files.

If you choose “no”, your identity will remain anonymous but your review may still be made public.

**Do you want your identity to be public for this peer review?**  For information about this choice, including consent withdrawal, please see our Privacy Policy

Reviewer #2: **Yes:** Kazunobu Okazaki

**********

# Editor report: Acceptance letter

**DOI:** 10.1371/journal.pone.0345346.r008

**Authors:** Hidenori Otani

PONE-D-26-10537R3

PLOS One

Dear Dr. Snape,

I'm pleased to inform you that your manuscript has been deemed suitable for publication in PLOS One. Congratulations! Your manuscript is now being handed over to our production team.

At this stage, our production department will prepare your paper for publication. This includes ensuring the following:

* All references, tables, and figures are properly cited

* All relevant supporting information is included in the manuscript submission,

* There are no issues that prevent the paper from being properly typeset

You will receive further instructions from the production team, including instructions on how to review your proof when it is ready. Please keep in mind that we are working through a large volume of accepted articles, so please give us a few days to review your paper and let you know the next and final steps.

Lastly, if your institution or institutions have a press office, please let them know about your upcoming paper now to help maximize its impact. If they'll be preparing press materials, please inform our press team within the next 48 hours. Your manuscript will remain under strict press embargo until 2 pm Eastern Time on the date of publication. For more information, please contact onepress@plos.org.

You will receive an invoice from PLOS for your publication fee after your manuscript has reached the completed accept phase. If you receive an email requesting payment before acceptance or for any other service, this may be a phishing scheme. Learn how to identify phishing emails and protect your accounts at https://explore.plos.org/phishing.

If we can help with anything else, please email us at customercare@plos.org.

Thank you for submitting your work to PLOS ONE and supporting open access.

Kind regards,

PLOS ONE Editorial Office Staff

on behalf of

Dr. Hidenori Otani

Academic Editor

PLOS One
